# Effects of Electrical Stimulation of NAc Afferents on VP Neurons’ Tonic Firing

**DOI:** 10.3389/fncel.2020.599920

**Published:** 2020-11-23

**Authors:** Martin Clark

**Affiliations:** Department of Psychology, The University of Central Lancashire, Preston, United Kingdom

**Keywords:** ventral pallidum, multielectrode, reward, substance P, nucleus accumbens

## Abstract

Afferents from the nucleus accumbens (NAc) are a major source of input into the ventral pallidum (VP). Research reveals that these afferents are GABAergic, however, stimulation of these afferents induces both excitatory and inhibitory responses within the VP. These are likely to be partially mediated by enkephalin and substance P (SP), which are also released by these afferents, and are known to modulate VP neurons. However, less is known about the potentially differential effects stimulation of these afferents has on subpopulations of neurons within the VP and the cellular mechanisms by which they exert their effects. The current study aimed to research this further using brain slices containing the VP, stimulation of the NAc afferents, and multi-electrode array (MEA) recordings of their VP targets. Stimulation of the NAc afferents induced a pause in the tonic firing in 58% of the neurons studied in the VP, while 42% were not affected. Measures used to reveal the electrophysiological difference between these groups found no significant differences in firing frequency, coefficient of variation, and spike half-width. There were however significant differences in the pause duration between neurons in the dorsal and ventral VP, with stimulation of NAc afferents producing a significantly longer pause (0.48 ± 0.06 s) in tonic firing in dorsal VP neurons, compared to neurons in the ventral VP (0.21 ± 0.09 s). Pauses in the tonic firing of VP neurons, as a result of NAc afferent stimulation, were found to be largely mediated by GABA_A_ receptors, as the application of picrotoxin significantly reduced their duration. Opioid agonists and antagonists were found to have no significant effects on the pause in tonic activity induced by NAc afferent stimulation. However, NK-1 receptor antagonists caused significant decreases in the pause duration, suggesting that SP may contribute to the inhibitory effect of NAc afferent stimulation *via* activation of NK-1 receptors.

## Introduction

The ventral pallidum (VP) is a major output structure of the striatum and is innervated by afferents from the nucleus accumbens (NAc). Many consider these afferents to be the main input of the VP (Haber et al., 1985; Bolam et al., 1986). Activation of these afferents is involved in pleasurable responses and sensitization to drugs of abuse (Smith and Berridge, 2005; Creed et al., 2016). These afferents affect VP neurons through the release of associated neurotransmitters and peptides. The NAc afferents of the VP release GABA (Walaas and Fonnum, 1979; Mogenson et al., 1983; Yang and Mogenson, 1985; Churchill et al., 1990; Reiner and Anderson, 1990; Zaborszky and Cullinan, 1992; Churchill and Kalivas, 1994; Kitamura et al., 2001). However, research has shown that stimulation of these afferents produces both inhibition and excitation of VP neurons (Chrobak and Napier, 1993). Enkephalin and substance P (SP), as well as GABA, is probably involved in these responses as both are known to be released by these afferents (Haber et al., 1985; Napier et al., 1995; Lu et al., 1997). Enkephalin, has been shown to contribute (along with GABA) to the inhibition of VP neurons seen in response to stimulation of NAc afferents (Napier et al., 1992), and SP is known to produce excitatory responses in the VP (Napier et al., 1995), therefore SP is a likely candidate to be involved in the excitation of VP neurons as a result of NAc afferent stimulation.

Questions remain as to what neurons the afferent connections of the NAc target in the VP. Research suggests they may directly target cholinergic interneurons in the VP (Grove et al., 1986; Zaborszky et al., 1986; Zaborszky and Cullinan, 1992), although recent research by Root et al. (2015) suggest NAc afferents also directly innervate GABAergic neurons of the VP. Further research needs to elucidate how stimulation of these afferents modulates the VP neurons they innervate. The question also remains as to what receptors are involved in the modulatory effects of NAc inputs to the VP. The research suggests a strong involvement of GABA_A_ receptors (Chrobak and Napier, 1993). However NK-1 receptors (Maeno et al., 1993; Chen et al., 2001), δ-opioid receptors, and μ-opioid receptors (Lahti et al., 1989; Mitrovic and Napier, 1995) are also known to be present in the VP and neurons in the VP are responsive to their activation (Chrobak and Napier, 1993; Napier et al., 1995). It can be hypothesized that SP and enkephalin, released by NAc afferents to the VP target these receptors and modulate the effects of GABA on these neurons.

It is important to understand further how activation of NAc inputs into the VP, and the neuromodulators released, impact on VP neurons. Unraveling the potentially divergent, modulatory effects these connections have on the interneurons and projection neurons of the VP may reveal important information relating to drugs of abuse, affective disorders, and aspects of reward learning, as these areas, and their connections, are heavily involved in these processes.

## Materials and Methods

### Animals

Extracellular *in vitro* recordings were obtained from C57 mice that were bred and housed in the Biological services facility at the University of Sheffield.

### Slice Preparation

Mice of both sexes aged between 28 and 42 days were culled by cervical dislocation with death being confirmed by decapitation in line with the 1986 Animals (Scientific procedures) Act, and with approval from the UK Home Office. Following decapitation, the brain was rapidly removed from the skull and parasagittal slices of 400 μm obtained using a vibrating microtome (as per Beurrier et al., 2006; Cambden instruments) immersed in ice-cold (5°C), oxygenated (saturated 95% O_2_ and 5% CO_2_) sucrose cutting solution. This solution was prepared fresh daily and contained (in mM): Sucrose (184), KCl (2.5), NaH_2_PO_4_ (1.2), NaHCO_3_ (30), HEPES (20), Glucose (25), sodium ascorbate (5), Thiourea (2), sodium pyruvate (3), MgSO_4_ (10), CaCl_2_ (0.5).

Once cut, slices were transferred to a recovery chamber maintained at 26°C, containing a Tris recovery solution, which was continuously aerated with a carbogen mixture of 95% O_2_ and 5% CO_2_ gas. As with the sucrose cutting solution, the Tris recovery solution was freshly prepared daily and contained (in mM): Tris HCl (76), Tris base (19.5), KCl (2.5), NaH_2_PO_4_ (1.2), NaHCO_3_ (30), HEPES (20), Glucose (25), sodium ascorbate (5), Thiourea (2), sodium pyruvate (3), MgSO_4_ (10), CaCl_2_ (0.5) The slices incubated in this chamber for 30 min, before being transferred to another chamber for storage. This storage chamber was also maintained at 26°C and contained standard aCSF, continuously aerated with a carbogen mixture of 95% O_2_ and 5% CO_2_ gas. The standard aCSF was also made up daily and contained (in mM): NaCl (124), KCl (3), NaH_2_PO_4_ (1.2), NaHCO_3_ (26), Glucose (15), MgSO_4_ (2), CaCl_2_ (2). Slices were then left for a minimum of 60 min to equilibrate and recover before being transferred into the pMEA (perforated Multi-Electrode Array) recording chamber for electrophysiological recordings.

### pMEA Electrophysiological Recordings

Neural network activity was monitored and recorded using a pMEA (Multi-Channel Systems, Reutlingen, Germany). The pMEA contained 60 embedded electrodes, which were constructed of titanium nitrite. Each electrode has a diameter of 30 μm and they are spaced at 200 μm. Selected channels of electrical activity were recorded and digitized at a sampling rate of 10 kHz using a MEA1060-Up-BC amplifier and MC Rack software (version: 4.6.2, Multi-Channel Systems, Reutlingen, Germany).

Once in the recording chamber of the pMEA slices were moved into position over the electrodes of the pMEA using a fine-tipped artists brush. The bottom flow was then switched on to produce suction and fix the slice into position. Using a pair of fine-tipped tweezers, a mesh harp was then placed on top of the slice and a top flow applied as quickly as possible. This was done to ensure the health and viability of the slices. Both the top and bottom flow (perfusion) were of continuously aerated aCSF containing (in mM): NaCl (124), KCl (3), NaH_2_PO_4_ (1.2), NaHCO_3_ (26), Glucose (15), MgSO_4_ (2), CaCl_2_ (2). The bottom flow rate was maintained at 0.65–1 ml/min and the top flow was maintained at 3–5 ml/min. The activity was monitored for at least 1 h before recordings commenced.

### Slice Visualization

To identify the correct area of the slice for the recording of VP neurons and subsequent stimulation of the NAc, the pMEA was placed in the amplifier, then using an Olympus BX51 microscope with a 4× lens, visualized *via* a Tucson digital microscope camera, which sent a live feed to a Viglen computer (4 gb of memory and an i5 processor) running IS capture software.

The VP was considered as any area ventral to the caudal anterior commissure and before the caudal edge of the rostral anterior commissure as it subdivides the striatum into its dorsal and ventral extents. The NAc was considered as any area directly ventral to the rostral extent of the anterior commissure and the left of the identified VP region. For stimulation experiments identification of the dorsal and ventral extents of the VP was necessary. The dorsal VP was defined as any electrode, located within the VP, which was within 500 μm of the caudal anterior commissure. The ventral VP was defined as any region, located in the identified VP, over 600 μm away from the ventral edge of the caudal anterior commissure.

### Stimulation Methods and Protocols

Stimulation was performed using an STG 1002 (Multi-Channel Systems, Reutlingen, Germany), which was programmable *via* MC_stimulus software (version 2.1.5, Multi-Channel Systems, Reutlingen, Germany). Stimulation was delivered *via* the internal electrodes of the multi-electrode array (MEA) chip, the electrode to be used for stimulation was selected based upon the location of the slice in the MEA chip chamber, which was observed *via* the video stream from the camera mounted on the optic of the microscope. Electrodes were always chosen for stimulation that was at least 400 μm away from the rostral edge of the identified VP. Once the correct area, and the corresponding electrodes in this area, were identified, MC_select software (version 1.3.0, Multi-Channel Systems, Reutlingen, Germany), which allows you to allocate the embedded electrodes as stimulating channels, was used to administer a biphasic stimulation. Two electrodes adjacent to one another in the MEA were always selected for the delivery of stimulation. The stimulation was always bipolar, with a negative followed by positive polarity, in line with multichannel systems suggestions (Wagenaar et al., 2004, 2005). The voltage of stimulation never exceeded ±3,000 mV and lasted for a duration of:

• one stimulation of 200 μs duration followed by a 10-s gap, before the next trial.
• HFS (high-frequency stimulation) which was at 100 Hz: five stimulations of 300 μs duration with 9,700 μs gap between each, giving a total stimulation time of 50 ms. Each trial was 10 s apart.

### Pharmacology

All drugs were obtained from either Tocris Biosciences (UK) or Sigma Aldrich and were bath applied into the header reservoir feeding the top perfusion flow of the pMEA at the following concentrations:

1. Picrotoxin: (picrotoxin) 20 μM.
2. *N*-Acetyl-L-tryptophan 3, 5-*bis*(trifluoromethyl)benzyl ester: (L732,138) 20 μm.
3. (5α)-4, 5-Epoxy-3, 14-dihydro-17-(2-propenyl)morphinan-6-one hydrochloride: (Naloxone) 20 μM.
4. [D-Ala^2^, NMe-Phe^4^, Gly-ol^5^]-enkephalin: (DAMGO) 20 μM.

### Data Analysis

Data was acquired using Mc_rack software (version: 4.6.2) and a MEA1060-Up-BC amplifier (Multi-Channel Systems, Reutlingen, Germany). Files (.mcd) were converted to .ced files using multichannel data manager software (version: 1.9.7, Multi-Channel Systems, Reutlingen, Germany) for off-line analysis using Spike 2 software (C.E.D). Please see Clark and Bracci (2018) for further detail on how this software was used for offline spike sorting.

See figure legends for information on result expression. All error bars are expressed as SEM.

Pause duration was calculated as the amount of time taken for the neuron to return to 90% of the pre-stimulation firing rate. If this occurred in <100 ms these neurons were considered as non-pauses. If the pause duration was >100 ms these neurons were considered pauses. This was done to ensure the HFS, which lasted for 50 ms, was not falsely accepted as a pause in firing.

To assess differences in a neuron’s firing frequency in different pharmacological treatments. I measured consecutive inter-spike intervals (ISIs) at the end of the stimulation protocol and during the final 60 s of each pharmacological condition. Average ISIs for relevant conditions were then compared using a Student’s *t*-test. A statistically significant difference was considered to be present if *P* < 0.05. If treatment caused a significant increase in ISI, I refer to this observation in the results as a significant decrease in firing frequency and an inhibitory effect of the treatment. If treatment caused a significant decrease in ISI, I refer to this observation as a significant increase in firing frequency and an excitatory effect of the treatment. The coefficient of variation and spike half-width was also calculated in different pharmacological treatments during the final 60 s of each pharmacological condition. The coefficient of variation (CoV) was calculated as a measure of spike train variability in different pharmacological conditions and as a potential way of identifying different neuronal types in the VP. It was calculated as the standard deviation (ISI)/Mean ISI. The threshold for spike detection was considered to be reached when the recorded voltage departed from baseline (0 mV in AC recording mode) by more than the standard deviation of the voltage recorded for that channel during an apparently quiescent period (of at least 3 s). Spikes consisted of a biphasic negative-positive waveform. Spike amplitude was defined as the difference between the negative voltage peak and the spike threshold level defined above. Spike half-width was defined as the time the value of the recorded voltage (measured from the threshold level) remained more negative than half of the spike amplitude (Pettersen and Einevoll, 2008; Clark and Bracci, 2018).

## Results

### Stimulation of the NAc Inhibits Tonic Firing in Some VP Neurons

To explore the effect that NAc afferents have on VP neurons, I stimulated the NAc every 10 s and measured the changes in tonic firing activity within the dorsal and ventral extents of the VP.

**Figure 1:**
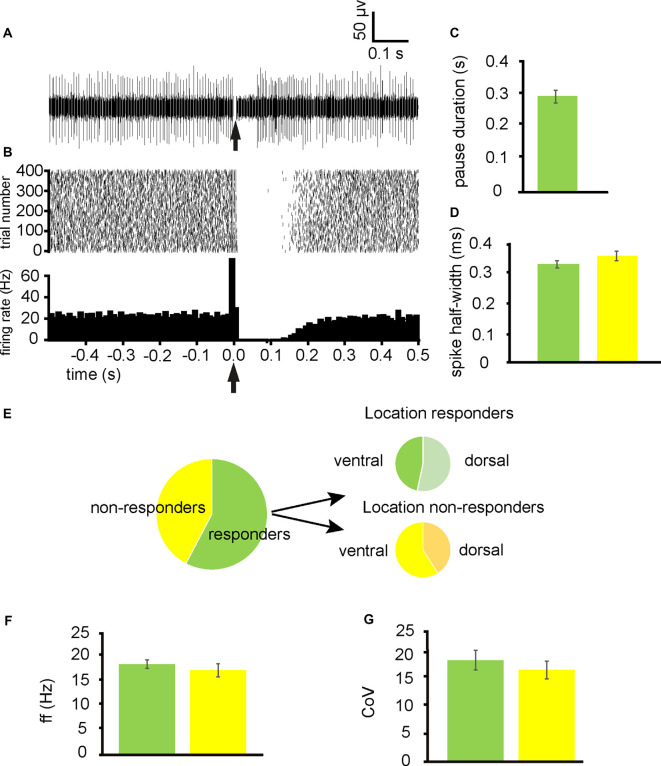
Stimulation ofthe nucleus accumbens (NAc) results in inhibition of tonic firing in some ventral pallidum (VP) neurons. All experiments represented in this figure used the second stimulation protocol (100 Hz) identified in the “Materials and Methods,” section. **(A)** A typical example of a raw data trace from a neuron in the VP with a pause (responder) in tonic activity after stimulation of the NAc. **(B)** A typical example raster plot (above) and peri-stimulus time histogram (PTSH, below, in bins of 10 ms) of tonic firing activity in a VP neuron 0.5 s pre-stimulation and 0.5 s post-stimulation of the NAc. The raster plot exemplifies one neuron response during 400 applications of stimulation. **(C)** Bar chart representing average pause duration for those neurons that were responders to NAc stimulation. **(D)** Bar chart representing the non-significant difference in spike half-width (ms) for neurons that responded to NAc stimulation (green) and those that were non-responders (yellow). **(E)** Pie charts representing the proportion of neurons identified in the VP for analysis that responded to NAc stimulation, and the proportions that were located ventrally vs. dorsally within the VP. **(F)** Bar chart representing non-significant differences in baseline firing frequency rates for neurons that responded to NAc stimulation (green) and those that were non-responders (yellow). **(G)** Bar chart representing the non-significant difference in baseline coefficient of variation for those neurons that responded to NAc stimulation (green) and those that were non-responders (yellow).

From 13 experiments, 52 neurons were identified for analysis as they had a stable tonic firing rate after 1 h of slice accommodation in the multielectrode array. Stimulation of the NAc induced a pause (referred to as responders) in firing in 30/52 of these neurons (Figures 1A,B), with 53% being located in dorsal extents of the VP and 47% being located in ventral extents of the VP (Figure 1E). The 22/52 were not affected by NAc stimulation (referred to as non-responders) with 41% being located in the dorsal extent of the VP and 59% being located in the ventral extent of the VP (Figure 1E). Pause duration was 0.29 ± 0.03 s for those classed as responders (Figure 1C).

To ascertain if pauses were elicited in different types of neurons within the VP, firing frequency, coefficient, and variation and spike half-widths were calculated for responders and non-responders. There was no significant (*P* > 0.05) difference in the average baseline firing frequency rates between responders 18.16 ± 3.16 Hz and non-responders 15.27 ± 2.83 Hz (Figure 1F). There was no significant (*P* > 0.05) difference in the coefficient of variation between responders 27.07 ± 9.61 and non-responders 19.27 ± 2.51 (Figure 1G). Finally, there was no significant difference in spike half-width (ms) between responders 0.32 ± 0.02 ms and non-responders 0.35 ± 0.03 ms (Figure 1D).

I conclude that a significant population of neurons in the VP is directly inhibited by afferents from the NAc and that these neurons are evenly dispersed between the dorsal and ventral extents of the VP. There is however a significant population that was not directly inhibited by NAc stimulation.

### NAc Stimulation Induces Pauses of Different Durations Dorsally Compared to Ventrally in the VP

To ascertain if there were any regional differences in pause duration between dorsal and ventral extents of the VP, the pause duration of neurons identified as in dorsal portions of the VP were compared to neurons in ventral portions of the VP.

**Figure 2:**
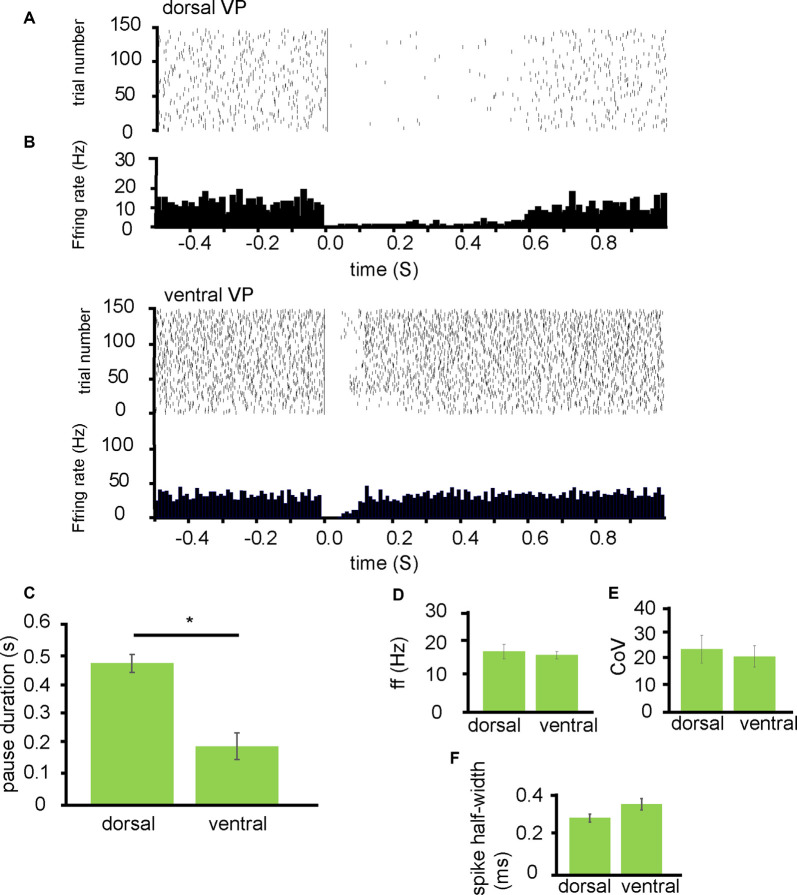
NAc stimulation induces a longer pause duration in dorsal VP neurons than ventral. All experiments presented in this figure used the first stimulation protocol (one stimulation of 200 μs duration followed by a 10-s gap) identified in the “Materials and Methods” section. **(A)** A typical example raster plot of tonic firing activity in a VP neuron 0.5 s pre-stimulation and 1.0 s post-stimulation of the NAc. The raster plot exemplifies one neuron’s response in the dorsal VP during 150 applications of stimulation. **(B)** A typical example raster plot of tonic firing activity in a VP neuron 0.5 s pre-stimulation and 1.0 s post-stimulation of the NAc. The raster plot exemplifies one neuron response in the ventral VP during 150 applications of stimulation. **(C)** Bar chart representing significant differences (*P < 0.05) in pause duration between those neurons studied in the dorsal VP compared to those in the ventral VP. **(D)** Firing frequency (Hz) for neurons that paused in response to NAc stimulation in the dorsal VP compared to those in the ventral VP. **(E)** Coefficient of variation for neurons that paused in response to NAc stimulation in the dorsal VP and those in the ventral VP. **(F)** Spike half-width (ms) for neurons that paused in response to NAc stimulation in the dorsal VP compared to those in the ventral VP.

From 10 experiments I identified 17 neurons in the VP for analysis as they responded to NAc stimulation. The 7/17 of these were classified as being located dorsally (Figure 2A) in the VP and 10/17 of these were classified as located ventrally within the VP (Figure 2B). Ventrally located neurons paused for an average duration of 0.21 ± 0.09 s while dorsally located neurons paused for an average duration of 0.48 ± 0.06 s. This was significantly different (*P* < 0.05; Figure 2C).

To ascertain if these differences represented different populations of neurons in the dorsal VP compared to the ventral VP I compared them based on the baseline firing frequency, coefficient of variation, and spike half-width profiles. There was no significant difference in the average baseline firing frequency rates between those neurons located dorsally within the VP 17.13 ± 4.06 Hz and those located ventrally within the VP 16.13 ± 2.08 Hz (Figure 2D). There was no significant difference in the coefficient of variation between those neurons located dorsally 24.85 ± 11.06 and those located ventrally 22.05 ± 8.53 (Figure 2E). Finally, there was no significant difference in spike half-width (ms) between those located dorsally 0.29 ± 0.04 ms and those located ventrally 0.36 ± 0.05 ms (Figure 2F).

I tentatively conclude that inputs arriving from the NAc to dorsal regions in the VP have a greater inhibitory effect on the VP’s tonic firing than those inputs from the NAc arriving in more ventral regions of the VP, but this does not appear to be related to the type of neuron they innervate within the VP.

### Picrotoxin Abolishes VP Neurons Inhibition by NAc Stimulation

Research suggests that afferent projections from the NAc innervating the VP are GABAergic. This should mean that the application of GABA_A_ antagonists should largely ameliorate the inhibition in tonic firing seen in response to NAc stimulation. To explore the role of GABA in the inhibition of VP neurons, the GABA_A_ antagonist picrotoxin was applied during stimulation of the NAc.

**Figure 3:**
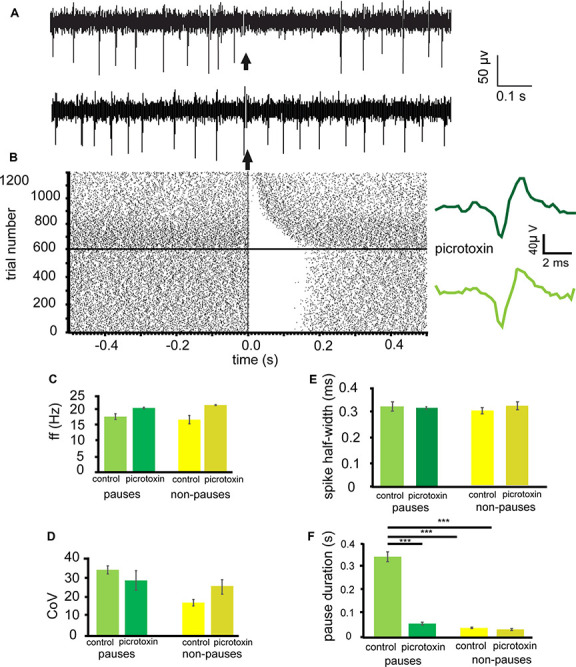
Picrotoxinsubstantially disinhibits VP neurons inhibited by NAc stimulation. All experiments presented in this figure used the first stimulation protocol (one stimulation of 200 μs duration followed by a 10-s gap) identified in the “Materials and Methods” section. ** (A)** A typical example of a raw data trace from a neuron in the VP with a pause in tonic activity after stimulation of the NAc and a raw trace from the same neuron after the application of picrotoxin with no pause in tonic activity. **(B)** A typical example raster plot of tonic firing activity in a VP neuron from 0.5 s pre-stimulation to 0.5 s post-stimulation of the NAc. The raster plot exemplifies one neuron response during 600 applications of stimulation in control conditions and 600 stimulations after the application of picrotoxin. To the right of the raster plot are two representative traces, the light green being before the application of picrotoxin and the dark green being after the application of picrotoxin. **(C)** Bar chart representing the no-significant difference in baseline firing frequency (Hz) for neurons that pause and those that did not pause in response to NAc stimulation, in control conditions and in the presence of picrotoxin. **(D)** Bar chart representing no-significant differences in baseline coefficient of variation for neurons that pause and those that did not pause in response to NAc stimulation, in control conditions, and in the presence of picrotoxin.** (E)** Bar chart representing no-significant differences in spike half-width for neurons that pause and those that did not pause in response to NAc stimulation, in control conditions, and in the presence of picrotoxin. **(F)** Bar chart representing significant differences (***P < 0.001) in pause duration for those neurons that paused in response to NAc stimulation (green) compared to the same neurons in the presence of picrotoxin, and compared to those neurons that did not pause in response to stimulation (yellow) in control conditions and in the presence of picrotoxin.

From 12 experiments 26 neurons in the VP were identified for analysis that paused in response to NAc stimulation (Figures 3A,B). The average pause duration for these 26 neurons was 0.34 ± 0.04 s. For these 26 neurons, after the application of picrotoxin, the pause duration was 0.06 ± 0.01 s (Figure 3F). As the pause duration measured after the application of picrotoxin was significantly (*P* < 0.001) shorter than before the application of picrotoxin (Figure 3E), I can conclude that the inhibition induced in tonically active VP neurons by NAc stimulation is largely a result of GABA release and subsequent activation of GABA_A_ receptors.

To investigate if the application of picrotoxin had any effect on electrophysiological characteristics of the VP neurons I measured baseline firing frequency rates, coefficient of variation, and spike half-width for the VP neurons pre and post-application of picrotoxin. The firing frequency (Hz) rates were not significantly (*P* > 0.05) different for any (pauses and non-pauses) VP neurons studied in the presence of picrotoxin 21.52 ± 0.49 Hz compared to control conditions 18.59 ± 1.97 Hz (Figure 3C). The coefficient of variation was also not significantly (*P* > 0.05) different for any (pauses and non-pauses) VP neurons studied in the presence of picrotoxin 29.75 ± 10.42 compared to control conditions 35.39 ± 4.34 (Figure 3D). Spike half-width (ms) was also not significantly (*P* > 0.05) different for any (pauses and non-pauses) VP neurons studied in the presence of picrotoxin 0.31 ± 0.01 ms compared to control conditions 0.32 ± 0.04 ms (Figure 3E).

I can therefore conclude that application of picrotoxin has no significant (*P* > 0.05) effects on the electrophysiological characteristics of the VP neurons but abolishes the inhibitory influence stimulation of NAc neurons has on VP neurons.

### Inhibition of VP Neurons by NAc Stimulation Is Not Affected by Opioid Antagonists

To investigate the role of opioids and opioid receptors in the inhibition of VP neurons by NAc stimulation, opioid agonists (DAMGO), and the non-specific opioid receptor antagonist, naloxone, were applied during stimulation of the NAc.

**Figure 4:**
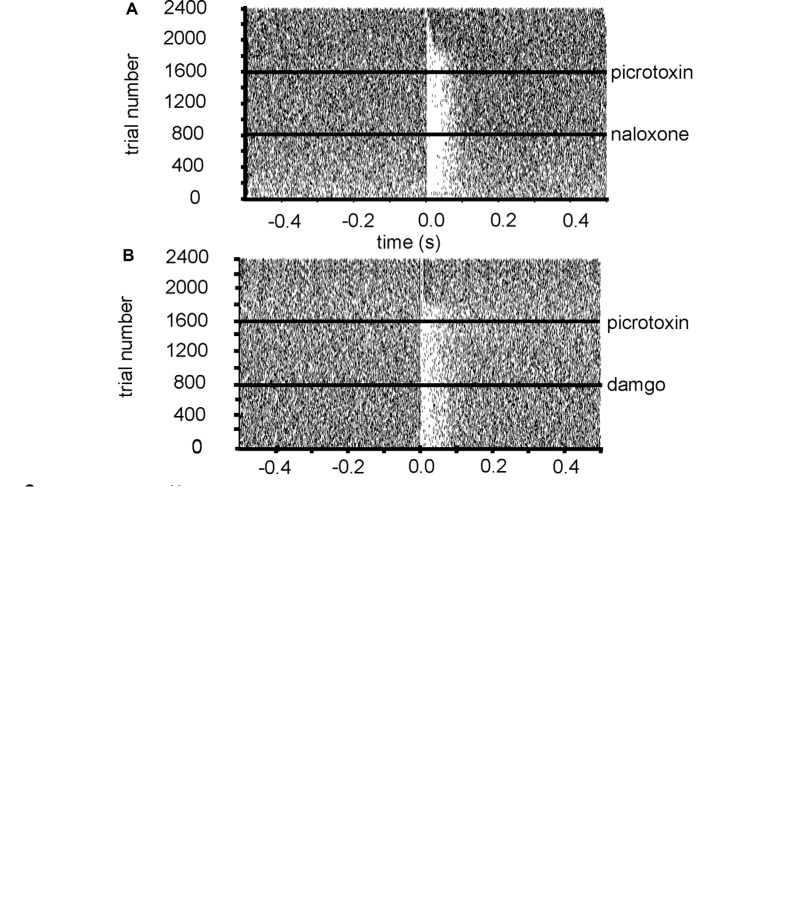
Inhibition of VP neurons by NAc stimulation is not affected by opioid agonists. All experiments presented in this figure used the first stimulation protocol (one stimulation of 200 μs duration followed by a 10-s gap) identified in the “Materials and Methods” section. **(A)** A typical example of a raster plot of tonic firing activity in a VP neuron (from −0.5 s to 0.5 s post-stimulation of the NAc). The raster plot shows a neuron’s response during 800 applications of stimulation in control conditions, 800 stimulations after the application of naloxone, and 800 stimulations after the application of picrotoxin. **(B)** A typical example raster plot of tonic firing activity in a VP neuron 0.5 s pre-stimulation and 0.5 s post-stimulation of the NAc. The raster plot exemplifies one neuron’s response during 800 applications of stimulation in control conditions, 800 stimulations after the application of DAMGO, and 800 stimulations after the application of picrotoxin. **(C)** Bar chart representing significant differences (**P < 0.01) in pause duration for neurons in control conditions, the presence of naloxone and the presence of picrotoxin. **(D)** Bar chart representing significant differences (*P < 0.01 and *P < 0.05) in pause duration for neurons in control conditions, the presence of DAMGO, and the presence of picrotoxin. **(E)** Bar chart representing non-significant (*P* > 0.05) differences in the average firing frequencies for neurons in control conditions, in the presence of naloxone and in the presence of picrotoxin. **(F)** Bar chart representing non-significant (*P* > 0.05) differences in the coefficient of variation for neurons in control conditions, in the presence of naloxone and in the presence of picrotoxin. **(G)** Bar chart representing non-significant (*P* > 0.05) differences in the average firing frequencies for neurons in control conditions, in the presence of DAMGO and in the presence of picrotoxin. **(H)** Bar chart representing non-significant (*P* > 0.05) differences in the coefficient of variation for neurons in control conditions, in the presence of DAMGO, and in the presence of picrotoxin.

From five experiments 11 neurons were identified for analysis that paused in response to NAc stimulation (Figure 4A). In control conditions, the average pause duration for these 11 neurons was 0.32 ± 0.06 s. For these 11 neurons after the application of naloxone, the average pause duration was 0.31 ± 0.07 s. The pause duration after the application of naloxone was not significantly (*P* > 0.05) different from the pause duration before the application of naloxone (Figure 4C). However, the pause duration (consistent with Figure 3E) was significantly (*P* < 0.05) reduced, compared to control conditions and naloxone, after the application of picrotoxin 0.04 ± 0.07 s (Figure 4C). I, therefore, conclude that opioid receptors do not modulate the pause in firing seen in VP neurons, induced by stimulation of the NAc afferents.

To investigate if the application of naloxone had any effect on electrophysiological characteristics of the VP neurons, I measured baseline firing frequency rates and coefficient of variation for the VP neurons (Figures 4E,F). The firing frequency (Hz) rates were not significantly (*P* > 0.05) different for the VP neurons studied in the presence of naloxone 19.35 ± 2.91 Hz compared to control conditions 18.00 ± 3.02 Hz and the presence of picrotoxin 19.82 ± 3.75 Hz. The coefficient of variation was also not significantly (*P* > 0.05) different for the VP neurons studied in the presence of naloxone 40.72 ± 16.79 compared to control conditions 27.07 ± 9.52 and picrotoxin 51.32 ± 22.93.

To verify that opioids did not play a role in modulation of the pause duration induced by NAc stimulation, I also applied DAMGO during stimulation of the NAc.

From three experiments five neurons were identified for analysis that paused in response to NAc stimulation (Figure 4B). In control conditions, the average pause duration for these five neurons was 0.40 ± 0.11 s. For these five neurons after the application of DAMGO, the average pause duration was 0.31 ± 0.08 s (Figure 4D). The pause duration after the application of DAMGO was not significantly (*P* > 0.05) different from the pause duration before the application of DAMGO. The pause duration was, however significantly (*P* < 0.05) reduced, compared to control conditions and DAMGO, after the application of picrotoxin 0.04 ± 0.07 s (Figure 4D).

To check if DAMGO affected the electrophysiological characteristics of VP neurons I measured baseline firing frequency rates and coefficient of variation for the VP neurons (Figures 4G,H). The firing frequency (Hz) rates were not significantly (*P* > 0.05) different for the VP neurons studied in the presence of DAMGO 14.01 ± 4.26 Hz compared to control conditions 15.89 ± 3.79 Hz and picrotoxin 11.82 ± 1.76 Hz. The coefficient of variation was also not significantly (*P* > 0.05) different for the VP neurons studied in the presence of DAMGO 60.89 ± 12.69 compared to control conditions 39.95 ± 8.63 and picrotoxin 53.42 ± 10.21.

I, therefore, conclude that opioid receptors appear to have no modulatory effect on the pause in firing seen in VP neurons, induced by stimulation of the NAc afferents.

### NK-1 Receptors Modulate the Effect of NAc Stimulation on VP Tonic Firing

To investigate the effects of SP receptors on the inhibition of VP neurons by NAc stimulation, I applied L732,138, which is an NK-1 receptor antagonist, during stimulation of the NAc.

**Figure 5:**
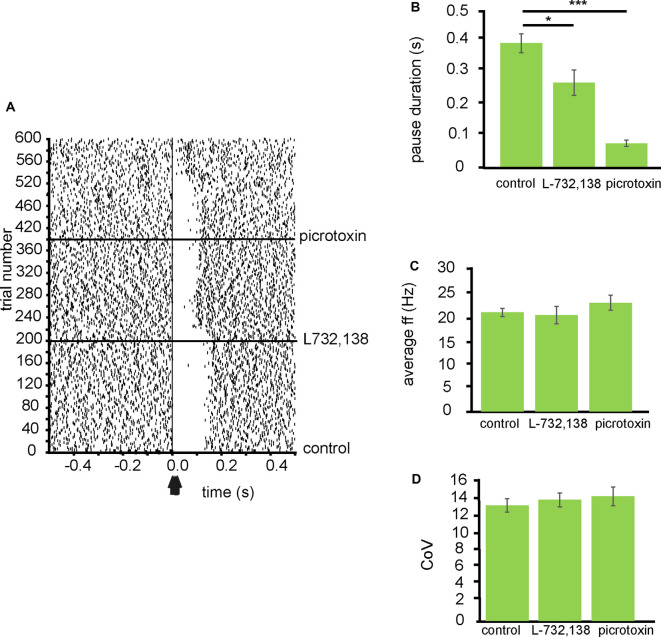
Inhibition of VP neurons by NAc stimulation is modulated by NK-1 receptors. All experiments represented in this figure used the second stimulation protocol (100 Hz) identified in the “Materials and Methods” section. **(A)** A typical example raster plot of tonic firing activity in a VP neuron from 0.5 s to 1.0 s post-stimulation of the NAc. The raster plot exemplifies one neuron response during 200 applications of stimulation in control conditions, 200 stimulations after the application of L732,138, and 200 stimulations after the application of picrotoxin. **(B)** Bar chart representing significant (*P < 0.05, ***P < 0.001) differences in the average pause duration (ms) for neurons in the VP after NAc stimulation. Each bar represents the average pause duration in different pharmacological conditions: in control conditions, in the presence of L732,138, and in the presence of picrotoxin. **(C)** Bar chart representing non-significant (*P* > 0.05) differences in the average firing frequencies for neurons in control conditions, in the presence of L732,138 and in the presence of picrotoxin. **(D)** Bar chart representing non-significant (*P* > 0.05) difference in the coefficient of variation for neurons in control conditions, in the presence of L732,138 and in the presence of picrotoxin.

From three experiments eight neurons were identified for analysis that paused in response to NAc stimulation (Figure 5A). In control conditions, the average pause duration for these neurons was 0.39 ± 0.06 s, for these eight neurons, after the application of L732,138 the average pause duration was 0.26 ± 0.06 s. The pause duration, after the application of L732,138, was significantly (*P* < 0.05) reduced compared to the pause duration in control conditions (Figure 5B). The pause duration was further significantly (*P* < 0.001) reduced, compared to control conditions, with the application of picrotoxin 0.07 ± 0.02 s (Figure 5B).

To investigate if the application of L732,138 had any effect on electrophysiological characteristics of the VP neurons I measured baseline firing frequency rates and coefficient of variation for the VP neurons. The firing frequency (Hz) rates were not significantly (*P* > 0.05) different for the VP neurons studied in the presence of L732,138 20.15 ± 3.67 Hz compared to control conditions 20.75 ± 1.69 Hz and the presence of picrotoxin 22.77 ± 3.15 Hz (Figure 5C). The coefficient of variation was also not significantly (*P* > 0.05) different for the VP neurons studied in the presence of L732,138 13.55 ± 1.57 compared to control conditions 12.90 ± 1.53 and picrotoxin 13.93 ± 2.9 (Figure 5D).

I, therefore, conclude that SP contributes to the inhibition seen in VP neurons in response to NAc stimulation by activation of NK-1 receptors.

## Discussion

The present research concludes that the MEA setup can be used successfully to study the connections between the striatum and its output nuclei. In this case, specifically between the NAc and the VP. I provide support, using a novel method, for previous research (Wang et al., 2014) suggesting that the NAc afferents of the VP have an inhibitory impact on VP neurons, as activation of the NAc afferents produced inhibition in tonic firing for several VP neurons. The current study also identified several, tonically active neurons, across all VP territories that were not inhibited by activation of afferents from the NAc. I tentatively conclude that activation of NAc afferents does not inhibit all the neurons in the VP and this may relate to a type of neuron not directly targeted by NAc afferents.

These findings also revealed no distinct difference in any of our electrophysiological measures (firing frequency, coefficient of variation, and spike half-width) for those neurons that responded to the activation of NAc afferents and those that were non-responders. The lack of electrophysiological differences between those neurons that responded to NAc stimulation and those that were non-responders may be masked by within-group variability. Research suggests NAc afferents target multiple different types of neurons in the VP, including GABAergic and cholinergic (Grove et al., 1986; Zaborszky, 1989; Root et al., 2015), therefore the electrophysiological measures used may be distorted by this within-group variability. Future research could use BAC transgenic animals with a GFP (green fluorescent protein) promoter for fluorescence in cholinergic neurons, to identify these in the slice.

I conclude that SP, released from NAc afferents into the VP, contributes to the pause duration produced as a result of NAc stimulation through activation of NK-1 receptors. This is unexpected as blocking a known excitatory influence was expected to prolong the pause duration.

## Difference in Pause Duration Dorsal to Ventral Within the VP

The current study has also shown that the inhibitory effects of NAc inputs of the VP are evenly dispersed, with a similar number of neurons being inhibited in dorsal regions of the VP as were inhibited in more ventral regions of the VP. This even distribution across the VP is also the case for the neurons that were not inhibited by the activation of NAc afferents. On the other hand, there was a significant difference in the duration of the inhibition between neurons in dorsal and ventral territories of the VP, with the dorsal regions exhibiting a significantly longer pause in firing in response to activation of the NAc afferents than did ventrally located neurons in the VP. This could relate to the differences in the functional roles of these territories or code an imbalance in projections from dorsal regions and from ventral regions to their prospective targets. Root et al. (2015) suggest that the connections between the nucleus accumbens shell (NAcS) and ventromedial VP are involved in the initiation of drug-seeking (identifying the conditions for drug use) while nucleus accumbens core (NAcC) to dorsolateral VP is involved in the continuation of drug-seeking (addiction). I, therefore, speculate that drugs of abuse alter this imbalance in pause duration between ventral and dorsal VP neurons resulting in a shift from initiation to a continuation of drug-seeking. Further research would be pertinent in slices from drug sensitized animals, to investigate if the difference in pause duration between dorsal and ventral VP territories, is altered by sensitization to drugs of abuse.

The fact that brain slice preparations are likely to only preserve a small proportion of the connections between brain regions, could account for the differences in pause duration dorsally to ventrally within the VP. Caution should be taken as these results could be due to more afferents to dorsal VP being intact in our brain slice preparations compared to the number of afferents to ventral VP.

### Neurotransmitters and Neuropeptides Modulating Pause Duration

As for the pharmacology of these inhibitory effects on VP neurons, I found that the GABA_A_ antagonists (picrotoxin) largely removed the pause in tonic firing, seen in some VP neurons, induced by activation of the NAc afferents. I conclude that the inhibitory effect of NAc afferent activation on a subset of VP neurons is predominantly mediated by the release of GABA and the activation of GABA_A_ receptors on target VP neurons, thus resulting in a pause in tonic activity. This makes sense in the light of the dominant labeling/expression of GABA_A_ as opposed to GABA_B_ in the VP (Zilles et al., 1991; Henderson, 1995). This also supports the immunohistochemical research (Churchill and Kalivas, 1994) and the previous electrophysiological work of Chrobak and Napier (1993), which suggests GABA antagonists disinhibit VP neurons. Although the pause duration we found was consistent with the published literature of evoked GABA potentials in the pallidal regions at room temperature 25°C (Ogura and Kita, 2000). Future research needs to confirm these likely GABA currents. This can be done by using patch-clamp techniques to record in the voltage-clamp mode, to confirm that a GABA current was indeed activated in VP neurons by stimulation of the NAc.

Multiple different subtypes of GABA_A_ receptors are known to be expressed in the VP and its dorsal division the GP (globus pallidus), including the α1β2γ2 receptor subtype (Duncan et al., 1995) and the α2β2γ2 subtype (Goetz et al., 2007). Immunocytochemistry studies (Pirker et al., 2000) show that many of the subunit variants of GABA_A_ receptors (α1–α6, β1–β3, γ1–γ3 and δ) are expressed in the VP, suggesting a large variety of GABA_a_ receptor types in the region. While the current study does not elucidate their relative contributions to the pause evoked by NAc stimulation, focused targeting of these specific subtypes would prove a fruitful line of inquiry for future research of the VP.

Recent research suggests both direct and indirect pathway MSN’s project into the VP (Creed et al., 2016; Kupchik and Kalivas, 2016). These release enkephalin (indirect) and SP (direct) and therefore these neuropeptides would seem likely candidates to contribute to the pause in tonic firing induced by activation of the NAc afferents. The current study found there to be no effect of opioid antagonists on the pause induced by stimulation of afferents from the NAc. I conclude that, although enkephalin may be released by these neurons, it has no apparent modulatory effect on the neurons directly inhibited by activation of these afferents. Opioid antagonists were also not found to have any significant effect on the firing frequency and coefficient of variation for those neurons that paused. This is contrary to much of the research, which suggests enkephalin modulates VP neurons (Chrobak and Napier, 1993; Napier and Mitrovic, 1999). Indeed, caution is needed in the interpretation of our results. The stimulation technique used in our studies on opioids is known to favor the release of GABA/glutamate and not neuropeptides, such as enkephalin. Therefore, the lack of modulatory effect seen in response to opioid antagonists could simply be an artifact of the stimulation protocol not resulting in neuropeptide release. However, Mitrovic and Napier (1995) have shown that a significant proportion of VP neurons do not respond to any opioid agonist. It could therefore be that those neurons directly inhibited by NAc afferents are those that are not modulated by enkephalin.

The VP is heavily innervated by multiple other regions, supplying dopaminergic, serotonergic, glutamatergic, and cholinergic input (Root et al., 2015). It is therefore worth considering the role that other inputs into the VP play in modulating the activity of neurons in the region. There are well known dopaminergic inputs from the VTA (ventral tegmental area) with research showing that dopamine can induce dichotomous (excitatory and inhibitory) responses in different populations of VP neurons (Clark and Bracci, 2018). There are also serotonergic inputs from the Dorsal Raphe Nuclei (DRN), with research showing that serotonin, like dopamine, can induce dichotomous responses in VP neurons (Bengtson et al., 2004). Certainly, the contribution of these other inputs, and the interplay between these and those from the NAc, would be an excellent avenue for future research, revealing a likely complex interplay and effect on the pause induced by NAc stimulation in the VP.

### Methodological Issues With Stimulation Protocols

The stimulation protocol used for the experiments involving opioid antagonists was probably biased against the release of neuropeptides, as it involved the use of a low-frequency stimulation, which is known to favor the release of GABA and glutamate and not neuropeptides, such as enkephalin (Purves, 2001). Further to this I also used the non-selective opioid antagonist Naloxone. This meant that other opioid receptors (kappa and delta), which are known to be present in the VP (Mitrovic and Napier, 1995; Olive et al., 1997), would have been inhibited, thus potentially masking the effects of enkephalin by also modulating the effects of dynorphin. Future research should include high-frequency stimulation protocols, such as that used in our experiments with NK-1 antagonists, and selective enkephalin antagonists to avoid these issues.

### Substance P Modulation of Pause Duration

The current study suggested, unexpectedly, that SP contributes to the inhibitory effect of NAc input activation. Pause duration of VP neurons in response to NAc stimulation was significantly reduced by the application of NK-1 receptor antagonists, suggesting that SP released from these NAc afferents contribute to the inhibitory effect of NAc afferent activation, *via* activation of NK-1 receptors. This contradicts previous research showing that SP increases the firing rate of VP neurons and SP antagonists block increases in firing rate seen in response to NAc stimulation (Mitrovic and Napier, 1998). However the research of Mitrovic and Napier (1998) was carried out *in vivo*, therefore it plausible that the discrepancy results from the fact that in the intact brain the VP receives several other external inputs that are silent in the brain slices and could be modulated presynaptically by SP. Despite our data showing NK-1 receptor antagonists reduced the duration of the inhibition, they were not found to have any significant effect on the baseline firing frequency or the coefficient of variation of the VP neurons, suggesting that there is little endogenous activation of these receptors in the VP in the absence of stimulation. Chen et al.’s (2001) findings suggest that the majority of NK-1 receptors found in the VP are localized to cholinergic neurons. These cells could be involved in the effects reported here. Alternatively, SP may have caused presynaptic facilitation of GABA release from NAc fibers, thus prolonging the GABA-induced pauses. Further experiments should be aimed at testing this intriguing hypothesis. Furthermore, it could be that SP also affects other tachykinin receptors in the VP, as NK-3 receptors are known to be found in the VP (Maeno et al., 1993; Shughrue et al., 1996) and this alters the effect seen as they were not blocked in the current study. Future research could target these receptors or repeat the current work with the application of NK-3 receptor antagonists.

### Implications

In the current study, I have shown a novel method for investigation of the basal ganglia connections, which can be used to collect large amounts of data in relatively short periods, and also provides the ability to stimulate regions of interest and measure the effects in other afferent regions, providing insights into the modulatory connections and circuitry of brain regions.

I also provide evidence to show that NK-1 receptors modulate the inhibition in tonic firing as a result of NAc stimulation, which provides an intriguing mechanism, that could contribute to our understanding of how SP application has reinforcing effects when applied to the VP (Nikolaus et al., 1999). I tentatively suggest that this may be due to its facilitatory effect on inhibition produced by the NAc afferents of the VP, and may modulate reinforcement related signals between the NAc and VP, by increasing the duration of inhibition exerted by these inputs on the VP.

## Data Availability Statement

The raw data supporting the conclusions of this article will be made available by the author, without undue reservation.

## Ethics Statement

The animal study was reviewed and approved by University of Sheffield ethical review and licensing committee.

## Author Contributions

MC designed and carried out experiments, analyzed the data, and wrote the manuscript.

## Conflict of Interest

The author declares that the research was conducted in the absence of any commercial or financial relationships that could be construed as a potential conflict of interest.

## References

[B1] Bengtson C. P. Lee D. J. Osborne P. B. (2004). Opposing electrophysiological actions of 5-HT on noncholinergic and cholinergic neurons in the rat ventral pallidum *in vitro*. J. Neurophysiol. 92, 433–443. 10.1152/jn.00543.2003 14960557

[B2] Beurrier C. Ben-Ari Y. Hammond C. (2006). Preservation of the direct and indirect pathways in an *in vitro* preparation of the mouse basal ganglia. Neuroscience 140, 77–86. 10.1016/j.neuroscience.2006.02.029 16580149

[B3] Bolam J. P. Ingham C. A. Izzo P. N. Levey A. I. Rye D. B. Smith A. D.. (1986). Substance P-containing terminals in synaptic contact with cholinergic neurons in the neostriatum and basal forebrain: a double immunocytochemical study in the rat. Brain Res. 397, 279–289. 10.1016/0006-8993(86)90629-3 2432992

[B5] Chen L. W. Wei L. C. Liu H. L. Qiu Y. Chan Y. S. (2001). Cholinergic neurons expressing substance P receptor (NK1) in the basal forebrain of the rat: a double immunocytochemical study. Brain Res. 904, 161–166. 10.1016/s0006-8993(01)02460-x 11516425

[B6] Chrobak J. J. Napier T. C. (1993). Opioid and GABA modulation of accumbens-evoked ventral pallidal activity. J. Neural Transm. Gen. Sect. 93, 123–143. 10.1007/BF01245342 8217051

[B7] Churchill L. Kalivas P. W. (1994). A topographically organized gamma-aminobutyric acid projection from the ventral pallidum to the nucleus accumbens in the rat. J. Comp. Neurol. 345, 579–595. 10.1002/cne.903450408 7962701

[B8] Churchill L. Dilts R. P. Kalivas P. W. (1990). Changes in γ-aminobutyric acid, μ-opioid and neurotensin receptors in the accumbens-pallidal projection after discrete quinolinic acid lesions in the nucleus accumbens. Brain Res. 511, 41–54. 10.1016/0006-8993(90)90223-x 2158856

[B9] Clark M. (2018). An electrophysiological investigation of the extrinsic modulation of ventral pallidum neurons by dopamine and serotonin. The University of Sheffield Thesis. Available online at: http://etheses.whiterose.ac.uk/22438/. Accessed September 25, 2019.

[B10] Clark M. Bracci E. (2018). Dichotomous dopaminergic control of ventral pallidum neurons. Front. Cell Neurosci. 12:260. 10.3389/fncel.2018.00260 30186117 PMC6113373

[B12] Creed M. Niels N. R. Chandra R. Lobo M. K. Lüscher C. (2016). Convergence of reinforcing and anhedonic cocaine effects in the ventral pallidum. Neuron 92, 214–226. 10.1016/j.neuron.2016.09.001 27667004 PMC8480039

[B13] Duncan G. E. Breese G. R. Criswell H. E. McCown T. J. Herbert J. S. Devaud L. L. (1995). Distribution of [^3^H]zolpidem binding sites in relation to messenger RNA encoding the alpha 1, beta 2 and gamma 2 subunits of GABA_A_ receptors in rat brain. Neuroscience 64, 1113–1128. 10.1016/0306-4522(94)00433-6 7753379

[B14] Goetz T. Arslan A. Wisden W. Wulff P. (2007). GABA_A_ receptors: structure and function in the basal ganglia. Prog. Brain Res. 160, 21–41. 10.1016/S0079-6123(06)60003-4 17499107 PMC2648504

[B15] Grove E. A. Domesick V. B. Nauta W. J. H.. (1986). Light microscopic evidence of striatal input to intrapallidal neurons of cholinergic cell group Ch4 in the rat: a study employing the anterograde tracer*Phaseolus vulgaris* leucoagglutinin (PHA-L). Brain Res. 367, 379–384. 10.1016/0006-8993(86)91623-9 3697714

[B16] Haber S. N. Groenewegen H. J. Grove E. A. Nauta W. J. (1985). Efferent connections of the ventral pallidum: evidence of a dual striato pallidofugal pathway. J. Comp. Neurol. 235, 322–335. 10.1002/cne.902350304 3998213

[B17] Henderson Z. (1995). Expression of GABAA receptor subunit messenger RNA in non-cholinergic neurons of the rat basal forebrain. Neuroscience 65, 1077–1086. 10.1016/0306-4522(94)00542-d 7617163

[B18] Kitamura M. Ikeda H. Koshikawa N. Cools A. R. (2001). GABA A agents injected into the ventral pallidum differentially affect dopaminergic pivoting and cholinergic circling elicited from the shell of the nucleus accumbens. Neuroscience 104, 117–127. 10.1016/s0306-4522(01)00053-7 11311536

[B19] Kupchik Y. M. Kalivas P. W. (2016). The direct and indirect pathways of the nucleus accumbens are not what you think. Neuropsychopharmacology 42, 369–370. 10.1038/npp.2016.160 27909323 PMC5143491

[B20] Lahti R. A. Mickelson M. M. Jodelis K. S. McCall J. M. (1989). Comparative neuroanatomical distribution of the κ and μ opioid receptors in guinea pig brain sections. Eur. J. Pharmacol. 166, 563–566. 10.1016/0014-2999(89)90377-4 2553438

[B21] Lu X. Y. Behnam Ghasemzadeh M. Kalivas P. W. (1997). Expression of D1 receptor, D2 receptor, substance P and enkephalin messenger RNAs in the neurons projecting from the nucleus accumbens. Neuroscience 82, 767–780. 10.1016/s0306-4522(97)00327-8 9483534

[B22] Maeno H. Kiyama H. Tohyama M. (1993). Distribution of the substance P receptor (NK-1 receptor) in the central nervous system. Brain Res. Mol. Brain Res. 18, 43–58. 10.1016/0169-328x(93)90172-l 7683074

[B23] Mitrovic I. Napier T. C. (1995). Electrophysiological demonstration of μ, δ and κ opioid receptors in the ventral pallidum. J. Pharmacol. Exp. Ther. 272, 1260–1270. 7891342

[B24] Mitrovic I. Napier T. C. (1998). Substance P attenuates and DAMGO potentiates amygdala glutamatergic neurotransmission within the ventral pallidum. Brain Res. 792, 193–206. 10.1016/s0006-8993(98)00130-9 9593891

[B25] Mogenson G. J. Swanson L. W. Wu M. (1983). Neural projections from nucleus accumbens to globus pallidus, substantia innominata and lateral preoptic-lateral hypothalamic area: an anatomical and electrophysiological investigation in the rat. J. Neurosci. 3, 189–202. 10.1523/JNEUROSCI.03-01-00189.1983 6822855 PMC6564585

[B26] Napier T. C. Mitrovic I. (1999). Opioid modulation of ventral pallidal inputs. Ann. N. Y. Acad. Sci. 877, 176–201. 10.1111/j.1749-6632.1999.tb09268.x 10415650

[B27] Napier T. C. Chrobak J. J. Yew J. (1992). Systemic and microiontophoretic administration of morphine differentially effect ventral pallidum/substantia innominata neuronal activity. Synapse 12, 214–219. 10.1002/syn.890120306 1481140

[B28] Napier T. C. Mitrovic I. Churchill L. Klitenick M. A. Lu X. Y. Kalivas P. W. (1995). Substance P in the ventral pallidum: projection from the ventral striatum and electrophysiological and behavioral cinsequences of pallidal substance P. Neuroscience 69, 59–70. 10.1016/0306-4522(95)00218-8 8637633

[B29] Nikolaus S. Huston J. P. Hasenöhrl R. U. (1999). Reinforcing effects of neurokinin substance P in the ventral pallidum: mediation by the tachykinin NK1 receptor. Eur. J. Pharmacol. 370, 93–99. 10.1016/s0014-2999(99)00105-3 10323256

[B30] Ogura M. Kita H. (2000). Dynorphin exerts both postsynaptic and presynaptic effects in the globus pallidus of the rat. J. Neurophysiol. 83, 3366–3376. 10.1152/jn.2000.83.6.3366 10848555

[B31] Olive M. Anton B. Micevych P. Evans C. Maidment N. (1997). Presynaptic versus postsynaptic localization of mu and delta opioid receptors in dorsal and ventral striatopallidal pathways. J. Neurosci. 17, 7471–7479. 10.1523/JNEUROSCI.17-19-07471.1997 9295393 PMC6573463

[B32] Pettersen K. H. Einevoll G. T. (2008). Amplitude variability and extracellular low-pass filtering of neuronal spikes. Biophys. J. 94, 784–802. 10.1529/biophysj.107.111179 17921225 PMC2186261

[B33] Pirker S. Schwarzer C. Wieselthaler A. Sieghart W. Sperk G. (2000). GABA_A_ receptors: immunocytochemical distribution of 13 subunits in the adult rat brain. Neuroscience 101, 815–850. 10.1016/s0306-4522(00)00442-5 11113332

[B34] Purves D. (2001). Neuroscience. 4th Edn. Sunderland, MA: Sinauer Associates.

[B35] Reiner A. Anderson K. D. (1990). The patterns of neurotransmitter and neuropeptide co-occurrence among striatal projection neurons: conclusions based on recent findings. Brain Res. Rev. 15, 251–265. 10.1016/0165-0173(90)90003-7 1981156

[B36] Root D. H. Melendez R. I. Zaborszky L. Napier T. C. (2015). The ventral pallidum: subregion-specific functional anatomy and roles in motivated behaviors. Prog. Neurobiol. 130, 29–70. 10.1016/j.pneurobio.2015.03.005 25857550 PMC4687907

[B37] Shughrue P. J. Lane M. V. Merchenthaler I. (1996). *In situ* hybridization analysis of the distribution of neurokinin- 3 mRNA in the rat central nervous system. J. Comp. Neurol. 372, 395–414. 10.1002/(SICI)1096-9861(19960826)372:3<395::AID-CNE5>3.0.CO;2-Y 8873868

[B38] Smith K. S. Berridge K. C. (2005). The ventral pallidum and hedonic reward: neurochemical maps of sucrose “liking” and food intake. J. Neurosci. 25, 8637–8649. 10.1523/JNEUROSCI.1902-05.2005 16177031 PMC6725525

[B39] Wagenaar D. A. Madhavan R. Pine J. Potter S. M. (2005). Controlling bursting in cortical cultures with closed- loop multi- electrode stimulation. J. Neurosci. 25, 680–688. 10.1523/JNEUROSCI.4209-04.2005 15659605 PMC2663856

[B40] Wagenaar D. A. Pine J. Potter S. M. (2004). Effective parameters for stimulation of dissociated cultures using multi-electrode arrays. J. Neurosci. Methods 138, 27–37. 10.1016/j.jneumeth.2004.03.005 15325108

[B41] Walaas I. Fonnum F. (1979). The distribution and origin of glutamate decarboxylase and choline acetyltransferase in ventral pallidum and other basal forebrain regions. Brain Res. 177, 325–336. 10.1016/0006-8993(79)90783-2 497834

[B42] Wang L. Shen M. Yu Y. Tao Y. Zheng P. Wang F.. (2014). Optogenetic activation of GABAergic neurons in the nucleus accumbens decreases the activity of the ventral pallidum and the expression of cocaine-context-associated memory. Int. J. Neuropsychopharmacol. 17, 753–763. 10.1017/S1461145713001570 24456857

[B43] Yang C. R. Mogenson G. J. (1985). An electrophysiological study of the neural projections from the hippocampus to the ventral pallidum and the subpallidal areas by way of the nucleus accumbens. Neuroscience 15, 1015–1024. 10.1523/JNEUROSCI.18-13-05095.1998 4047397

[B44] Zaborszky L. (1989). Afferent connections of the forebrain cholinergic projection neurons, with special reference to monoaminergic and peptidergic fibers. EXS 57, 12–32. 10.1007/978-3-0348-9138-7_2 2533086

[B45] Zaborszky L. Cullinan W. (1992). Projections from the nucleus accumbens to cholinergic neurons of the ventral pallidum: a correlated light and electron microscopic double immunolabelling study in rat. Brain Res. 570, 92–101. 10.1016/0006-8993(92)90568-t 1617433

[B46] Zaborszky L. Heimer L. Eckenstein F. Leranth C. (1986). GABAergic input to cholinergic forebrain neurons: an ultrastructural study using retrograde tracing of HRP and double immunolabeling. J. Comp. Neurol. 250, 282–295. 10.1002/cne.902500303 3528237

[B47] Zilles K. Werner L. Qu M. Schleicher A. Gross G. (1991). Quantitative autoradiography of 11 different transmitter binding sites in the basal forebrain region of the rat–evidence of heterogeneity in distribution patterns. Neuroscience 42, 473–481. 10.1016/0306-4522(91)90390-a 1654535

